# Novelties for increased safety in cranio-vertebral surgery: a review

**DOI:** 10.1007/s00701-023-05769-2

**Published:** 2023-09-02

**Authors:** Enrico Tessitore, Ciro Mastantuoni, Ivan Cabrilo, Claudio Schonauer

**Affiliations:** 1 Department of Neurosurgery, Faculty of Medicine, Geneva University Hospital, Rue Gabrielle Perret Gentil 4, 1205 Geneva, Switzerland; 2 Department of Neurosurgery, Neurocenter of Southern Switzerland, Ente Ospedaliero Cantonale, Lugano, Switzerland; 3 Neurosurgery Unit A.O.R.N. “A.Cardarelli”, Naples, Italy

**Keywords:** Craniovertebral junction, Spine surgery, Biomaterials, Robotics, Navigation, Endoscopy, Customized implants

## Abstract

The cranio-vertebral junction (CVJ) was formerly considered a surgical “no man’s land” due to its complex anatomical and biomechanical features. Surgical approaches and hardware instrumentation have had to be tailored in order to achieve successful outcomes. Nowadays, thanks to the ongoing development of new technologies and surgical techniques, CVJ surgery has come to be widely performed in many spine centers. Accordingly, there is a drive to explore novel solutions and technological nuances that make CVJ surgery safer, faster, and more precise. Improved outcome in CVJ surgery has been achieved thanks to increased safety allowing for reduction in complication rates. The Authors present the latest technological advancements in CVJ surgery in terms of imaging, biomaterials, navigation, robotics, customized implants, 3D-printed technology, video-assisted approaches and neuromonitoring.

## Introduction

The cranio-vertebral junction (CVJ) has long been considered surgically challenging, due to its complex anatomical features that include vital neurovascular structures and to its unique biomechanical properties. Subsequently, high morbidity from short- and long-term complications was accepted and considered inevitable due to the complexity of surgery. However, the concept of “acceptable morbidity” is outdated as a result of surgical evolution, new technology, and patient awareness that provides the means and the expectations toward lesser morbidity in all fields of surgery. Lately, safety of CVJ surgery has increased due to the introduction and implementation of technological advancements, both in imaging and surgical assistance.

Anatomically, CVJ encompasses the occiput (C0) and the first two cervical vertebrae (C1 and C2) and so represents the transition zone between the skull and the mobile cervical spine. Its unique bony-ligamentous configuration allows for complex movements. While the C0-C1 joint is mostly involved in flexion and extension of the head, the C1-C2 joint provides most of the rotation in the cervical spine. Moreover, the configuration and orientation of the condyles and C1-C2 articular processes also enable lateral bending.

Surgically related considerations regarding CVJ anatomy include the following:

- The sharp angle between the occiput and the upper cervical spine creates a significant lever arm that counteracts surgical fixation devices [6]
- Space for the application of bone graft is limited compared to the thoracolumbar spine [64]
- Instrumentation is risk-laden due to the proximity of vascular and neural structures
- The weight discrepancy, especially in children, between the head and the cervical spine exposes the hardware to high loads and increases the risk of failure
- The thinness of the occipital bone usually present to the sides of the midline represents a challenge for occipital plating and may impact the hardware’s resistance to loading
- CVJ is a frequent site of bony and vascular anomalies (e.g., high-riding vertebral artery and os ponticulus) that increase the risk of hardware related complications.

Accordingly, instrumentation and surgical strategies have evolved to obtain more solid constructs while respecting the surrounding vital neurovascular structures. Various novel techniques—radiological, surgical, and technological—have been described in an attempt to enhance surgical efficacy. Here, we review the technological advancements of the last years and their implementation into modern surgical practice. Moreover, we analyzed their contribution to safety and effectiveness in the work-up and the surgical management of the cranio-vertebral junction. A deep knowledge of these novel techniques and of their implementation in clinical routine practice might make CVJ surgery safer and more effective than in the past.

## Novelties for safer CVJ surgery

Advancements in technology and knowledge have allowed increasing safety and reproducibility of surgical procedures. Patients harboring traumatic, tumoral, infective, degenerative and malformative lesions of the CVJ require very often complex surgeries that are associated with elevated neurological and functional risks.

Advancements concern imaging, biologics, customized implants and 3D-printed technology, intraoperative computer assistance (e.g., navigation and robotics), video-assisted approaches, and intraoperative neuromonitoring.

This article provides a critical review on the state of the art and the latest technological innovations involving safety in CVJ surgery.

### Advancements in diagnostic imaging

**Fig. 1:**
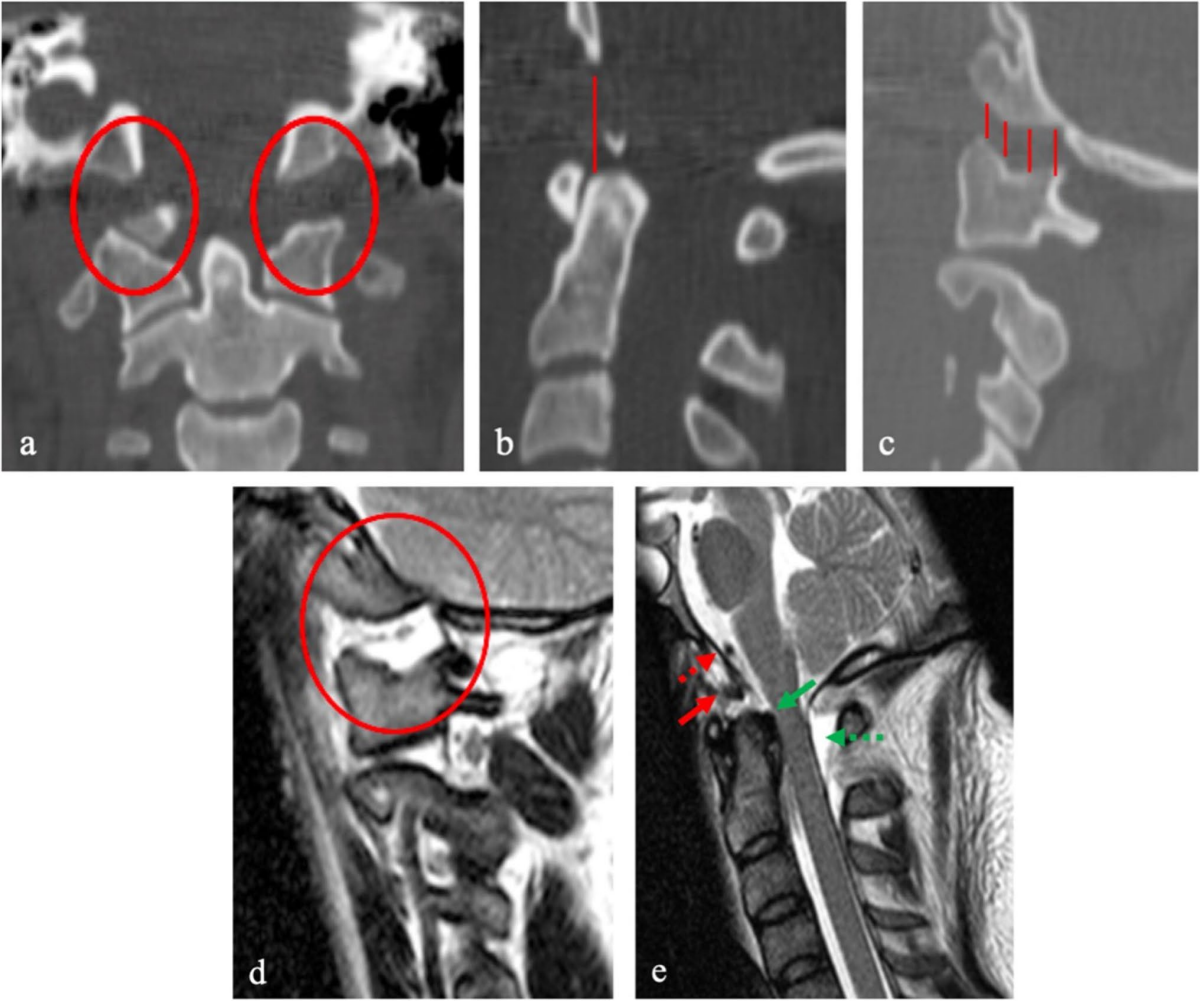
Atlantoaxial dislocation type II. **a** CT scan, coronal view, showing an increased occipital condyle-C1 interval (red circles). The condylar sum is defined as the sum of a patient’s left and right mean cranio-cervical interval (CCI) values. A distance > 2 mm in adults or > 5 mm in children, or a gross asymmetry between the two C0–C1 joints is highly sensitive and specific for AOD. **b** CT scan, sagittal view, focusing on basion-dens interval (red line). **c** CT scan, sagittal view, displaying an increased occipital condyle-C1 interval (red lines). **d** T2-weighted MRI, sagittal view, pointing out a fluid signal in the atlanto-occipital joint (red circle). **e** T2-weighted MRI, sagittal view, showing abnormal signal at the basion-dens interval, translating the disruption of the anterior atlanto-occipital membrane (red arrow), apical ligament (dotted red arrow), tectorial membrane (green arrow), and posterior atlantooccipital membrane (green dotted arrow)

Advancements in 3-dimensional (3D) CT-scan reconstruction and MR imaging have led to a better understanding and work-up of ligamentous injuries at CVJ, which, if undetected, might lead to a chronic painful instability [4]. Indeed, in the last decades, the gradual technological improvement has been followed by the introduction of novel diagnostic radiological criteria for atlantooccipital dislocation (AOD), as for example the condylar sum (Fig. 1a–c) [4, 15]. Historically, this life-threatening traumatic injury of the CVJ was diagnosed on X-rays or regular CT-scan. In current practice, MRI has increasingly come to complete this initial osseous radiological assessment by providing a targeted evaluation of the integrity of CVJ’s ligamentous structures [65].

In the current practice, STIR (short tau inversion recovery) sequences are particularly useful in this regard providing direct assessment of stability at the C1–C2 level, with potential implications on the therapeutic approach [68]. The transverse atlantal ligament (TAL) is crucial for C1–C2 instability. X-ray and CT-based measurements, such as the atlantodental interval (distance between posterior aspect of anterior tubercle of C1 and anterior aspect of odontoid peg) and the rule of Spence (where if the combined projection of the lateral masses of the atlas is more than 6.9 mm beyond the lateral masses of the axis, an injury to transverse ligament is likely), have lack of sensitivity [10]. In this scenario, Dickman et al. propose a new classification for TAL injuries based on X-ray, CT, and MR screening. The injuries were classified as disruptions of the substance of the ligament (type I) or as fractures and avulsions involving the tubercle for insertion of the TAL on the C1 lateral mass (type II), with type IA—namely, a central avulsion of the TAL—having no chance of healing under conservative treatment thus requiring fixation [12].

MRI is also useful to assess other ligamentous structures such as the apical and alar ligaments and the tectorial membrane or to identify indirect signs of latent craniovertebral instability in the trauma setting such as facet joint fluid effusion (Fig. 1d) or prevertebral hematomas (Fig. 1e) [65].

Finally, 3D CT-scan reconstruction and multiplanar reformatted (MPR) images provide a comprehensive overview of CVJ patho-anatomy. Innovative open-source software, such as Horos™ [7] or OsiriX™, enable surgeons 3D visualization in any projection and the possibility to crop out unwanted areas, thereby enhancing their understanding of the patho-anatomy and of neighboring neurovascular structures. The multiplanar reconstruction of CT images offers a more precise planning for screw trajectories, as standard orthogonal CT slices are not aligned to the screw path [45]. This shrewdness further reduces the misplaced rate a fortiori when applied on intra-operative CT scan.

**Fig. 2:**
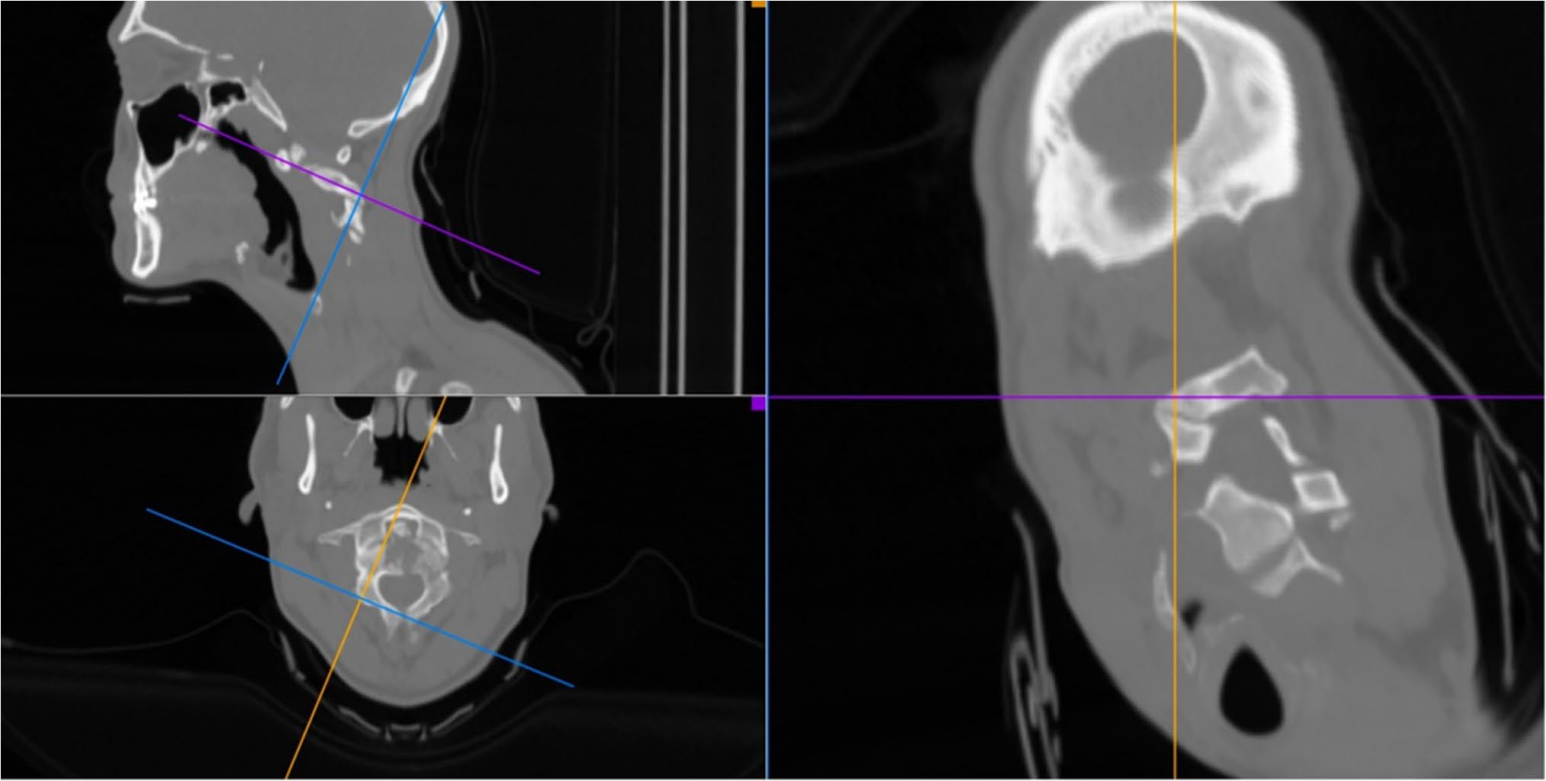
CT scan MPR reconstruction for planning screws trajectory using the open-source software Horos™

Additionally, it is important to underline that modern 3D reconstruction software can be used on laptops, enhancing the comfort and time spent for preoperative planning [76] (Fig. 2).

In our experience, the blooming of these rousing resources allows a more precise, tailored, and targeted treatment relying on the most advanced classifications and indications for CVJ diseases, reducing both overtreatment and undertreatment [26, 27, 33, 34, 65]. Finally, thanks to the current deployment of 3D CT-scan reconstruction and multiplanar reformatted (MPR) images, we perceive a better understanding of CVJ physiological and pathological anatomy leading to more thorough and satisfying diagnostic process and surgical planning. Moreover, the application of MPR to the intraoperative CT-scan enables a remarkable reduction in screw misplacement rate making CVJ surgery evermore safe thanks to the possibility to follow the screws all along their trajectory for assessing the presence of medial, lateral, or anterior pedicle wall violation [7, 71].

### Biologics

Occipitocervical fixation and atlantoaxial fixation are the mainstay techniques for CVJ fixation. The former is nowadays seldom used, being valuable chiefly for AOD or unstable condylar fractures. Contrariwise, the unfavorable tridimensional anatomy of C1–C2 and their close relationship with vital neurovascular structures led to the inception of several techniques in order to improve the safety and efficiency of C1–C2 fixation. Among those, the most employed worldwide are the Magerl technique, featured by transarticular C1–C2 screws, and the Harms-Goel technique, defined by the insertion of C1 lateral mass screws and isthmic C2 screws.

Against this backdrop, the fusion rate in CVJ fixation is hindered by the acute angle between C0 and C2, which acts as an unfavorable level arm and limits the space for bone grafting. The choice for the most appropriate bone material might depend on several factors, as economics, surgeons practice, and availability on the market.

Currently, used graft materials and biologics for spinal fusion include autografts from the iliac spine, occipital bone or C2 spinous process, and allografts obtained from cadavers and living donors, demineralized bone matrices (DBMs) [56], which are acid-extracted organic allografts with osteoinductive properties, ceramic grafts containing hydroxyapatite, and bone-morphogenetic proteins (BMPs), mesenchymal stem cells (MSCs), synthetic peptides, and autologous growth factors [14].

The iliac crest autograft used to be the standard-of-care for bone grafting due to its osteogenic, osteoinductive, and osteoconductive capabilities, while at the same time avoiding rejection by the immune system. However, it is associated with longer operating times, greater blood loss, postoperative walking difficulties, a increased infection risk, and donor site pain.

Allograft, frequently used in thoraco-lumbar and subaxial cervical spine procedures, has also been used in the upper cervical spine with encouraging results [50]. In the recent years, Zhang et al. [75], Godzik et al. [18], and Iyer et al. [34] compared autograft and allograft in atlantoaxial screws and rods fixation reporting similar fusion rates—about 100%—for both techniques, also in pediatric population with higher complications related to the autograft. Nevertheless, allograft did take longer to reach fusion when compared to autograft.

Recombinant human bone morphogenetic protein 2 (Rh-BMP2) is currently used to bolster fusion in spine procedures, while its use in CVJ surgery is still being investigated. Hood et al. [31], Ishida et al. [33], and Sayama et al. [60] reported occipitocervical and atlantoaxial fusion rates using rhBMP-2 of 100%, 94.3%, and 89.2%, respectively. Finally, in a cohort of 108 patients undergoing C1–C2 fixation, Guppy et al. found equal reoperation rates for symptomatic non-fusion between patients that received rh-BMP2 and autograft and those who received allograft alone [26].

In order to improve C1–C2 construct stability, intra-articular fusion is considered an effective alternative strategy, e.g. in irreducible atlantoaxial dislocation, in atlantoaxial instability, and in cases where posterior fusion cannot be carried out due to the absence of the C1 posterior arch or of C1–C2 distraction for basilar invagination, as described by Atul Goel [19–23]. Moreover, the use of bone graft augments the rigidity of the construct, while the cages increase intervertebral height [22, 38], providing a high rate of intra-articular fusion.

**Fig. 3:**
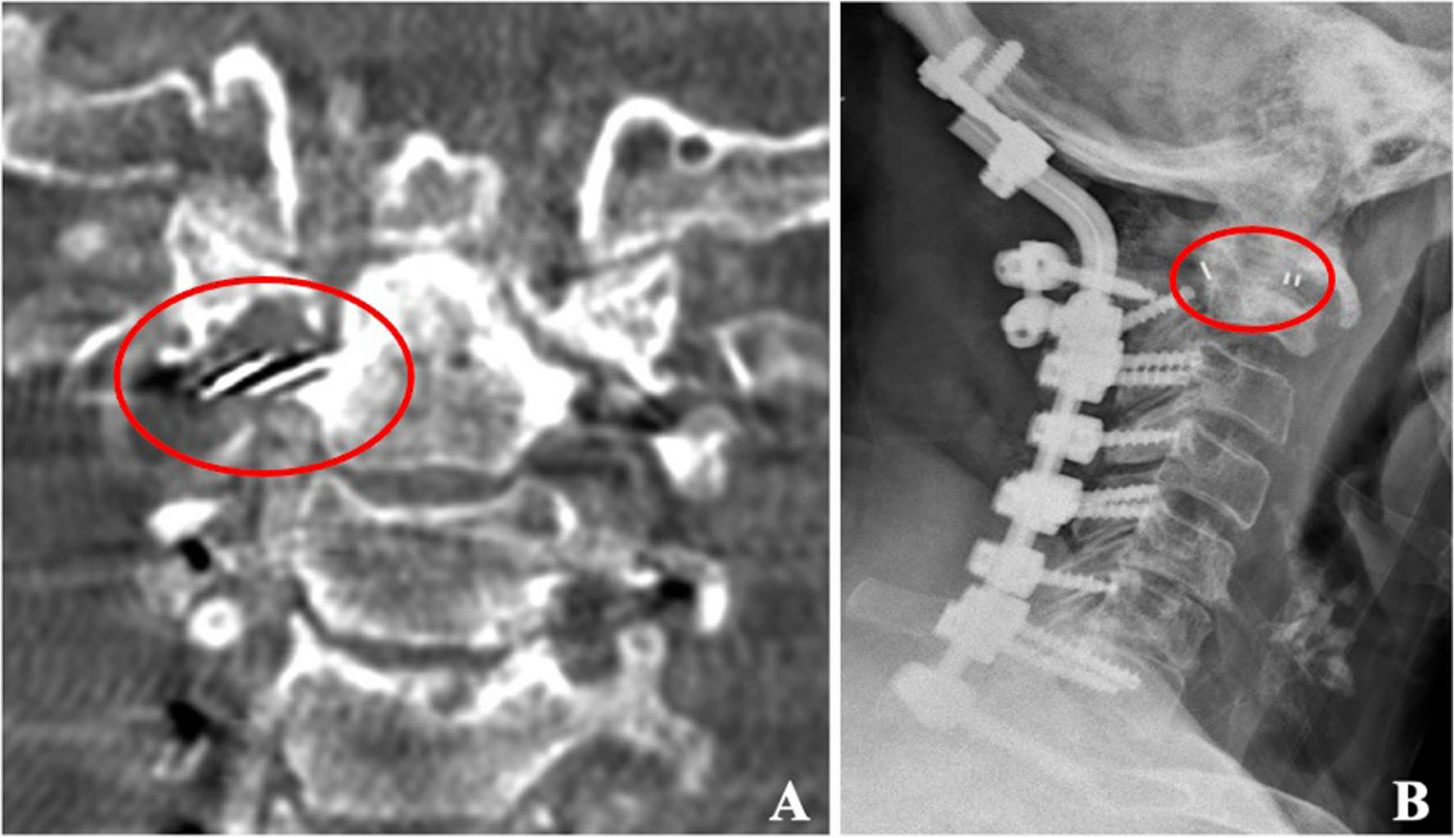
Implantation of unilateral right PEEK cage (red circles) between C1 and C2 articular processes to achieve vertical distraction and correction of basilar invagination via a posterior approach. **a** CT scan coronal view; **b** X-ray lateral view

In a cadaveric study, Polli et al. described the use of a modified vertebral body stent for the distraction of the C1–C2 joint [55], and in 2022, Rapisada et al. proposed the implantation of a trans-articular cage for C1–C2 distraction without the need to sacrifice the C2 nerve root. Authors have also implanted with success PEEK cages between C1 and C2 to achieve vertical distraction and correction of basilar invagination via a posterior approach (Fig. 3).

In this vein, we consider advancements in biomaterials the perfect synthesis of surgical strategy improvements, relying upon a deep insight into anatomy and physiopathology and bioengineering, whose innovation constantly support and bolster medical novelties. The epitome of this paradigm is the Goel-Harms technique that merges a thorough understanding of C1–C2 pathology and anatomy and the employment of innovative technologies [19–23]. Likewise, the quest for smarter and easier surgeries led to a paradigm shift toward the use of the allograft instead of autograft for CVJ fusion, with similar results in terms of fusion but remarkably better outcomes in terms of complications.

Although biomechanical stability is immediately provided by modern instrumentation (e.g., screws and rods), definitive stability is given by achievement of complete bony fusion around the hardware. Harrington introduced the universal concept that there is a “race between instrumentation failure and acquisition of spinal fusion” [28]. Modern biologics may accelerate fusion and reduce hardware failure rate preventing dangerous revision surgeries; nevertheless, there is still a long way to go for discovering a tool that will be able to overcome the limits of allograft, autograft, and recombinant proteins.

### Customized implants and 3D-printed technology

Customized implants are in line with the concept of personalized management strategies. Indeed, even before the popularization of 3D-printed technology, the need had been identified to adapt implants to the pathologic anatomy of patients. In more recent times, technological advances have allowed to respond to this need also for the CVJ, especially in the domain of spinal tumor [72]. Jeszenszky et al. proposed a custom-made C2 prosthesis that integrates an anterior transoral plate with a Harms titanium mesh cage and that allows removal of C2 while preserving C0–C1 motion [36]. In 2014, Ji et al. proposed a tailored titanium clivus plate to reconstruct the craniovertebral junction in combination with posterior fixation in order to augment upper cervical stabilization [37]. In 2022, Hu et al. proved that 3D printed vertebral bodies provided reliable reconstruction support comparable with titanium mesh after en bloc resection of C2 tumors [32]. Indeed, 3D technology has facilitated the process of tailoring such implants to a given patient and has also been reported to be helpful for surgical planning and teaching [8, 51].

In terms of patient-specific implants, 3D-printed prosthetic repair of bony defects has been recently applied to the CVJ. Xu et al. were among the first ones to report a case of upper cervical spine reconstruction using a 3D printed prothesis after a resection of Ewing sarcoma [72]. 4 years later, the same group described its experience using 3D printed vertebral body protheses in 9 cases of C2 spondylectomy for giant cell tumors, chordomas, Ewing sarcomas, paragangliomas, and hemangioendotheliomas. They molded the porous titanium scaffold implant based on a 1-mm thin slice CT-scan in a process that took 7 days. On follow-up, osteointegration was observed at the bone-implant contact surfaces in all patients [70].

In another example of patient-specific implants, Jian et al. reproduced the zygapophyseal joint of the cervical lateral mass using 3D printing in a surgical series that included two C2 chordomas [39].

The combined advances in biomaterials and deep biomechanical insight along with the 3D printing technology has led to the possibility of customized spinal implants bolstering the reconstruction process, especially for oncologic pathologies of CVJ [70]. Indeed, the irreplaceable mechanical role of the cranio-cervical junction may be seriously compromised by extensive tumor removal. Customized implants ensure improved stability of the junction while preserving mobility of adjacent segments [36, 37, 39]. In such conditions, surgeons may seek radical resection relying on advanced morphological and functional reconstruction techniques.

Finally, 3D models have proven to support the anatomic and surgical learning of the CVJ of residents and young surgeons [51]. Moreover, they can be useful in surgical planning for the more complex degenerative and dysplastic diseases or in cases of vascular anomalies [8].

Implementation of 3D implants improves safety thanks to the perfect congruence of the implant to the surgical target. Indeed, the surgical approach and dissection can be limited to the targeted structure (e.g., vertebral body in case of upper cervical vertebrectomy for tumors) reducing invasiveness of the surgical procedure and avoiding dangerous and unnecessary exposure of vital neurovascular structures.

### Navigation and robotics

The accuracy of screw placement is a main concern for spine surgeons since the introduction of transpedicular instrumentation techniques. In the current surgical practice, the development of computer-assisted screw insertion has reduced the rate of misplaced implants. Accordingly, navigation systems have also successfully been implemented in CVJ surgery [25, 41, 62, 63] and have been associated with a decrease in the incidence of implant-related neural and vascular injury. Undeniably, the risk of vertebral artery injury with conventional C-arm guidance has been reported to be as high as 8–9.5% [62, 63], with rates decreasing to 0.3–2% [40] with the use of the navigation and intraoperative CT-scan. Additionally, Hitti et al. have shown a reduction in surgical time and blood loss using navigation during atlantoaxial fixation [29].

**Fig. 4:**
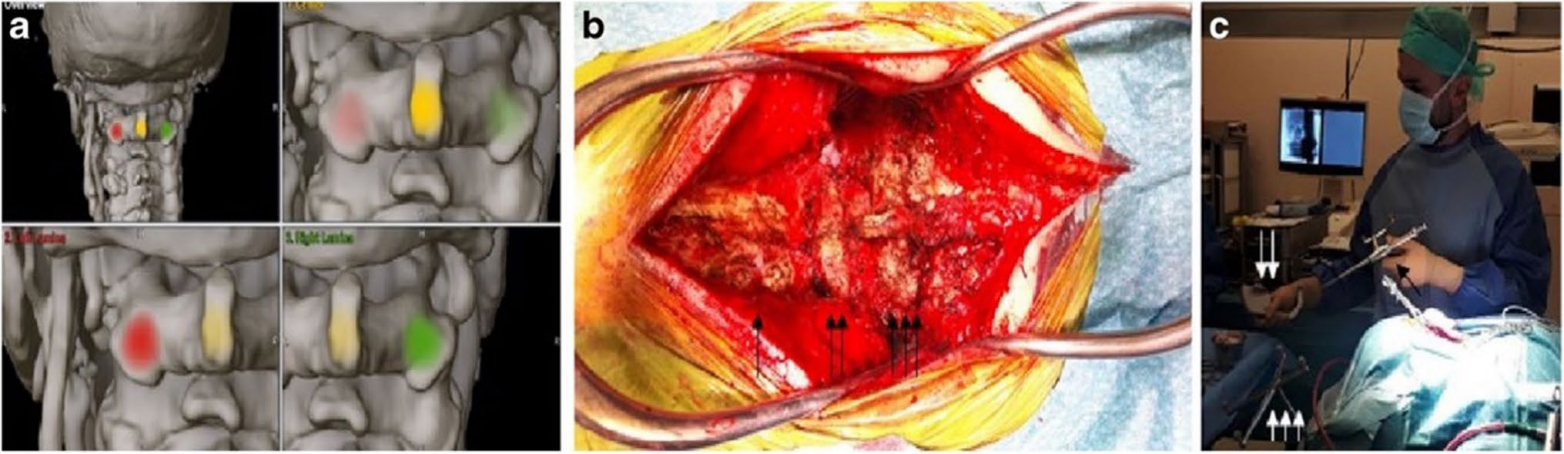
**a** Navigation registration with surface matching technique. **b** Intra-operative view of the exposed C0 (arrow), C1 (double arrow), and C2 posterior elements (triple arrow). **c** Intraoperative calibration of the navigated drill guide (arrow) using the dedicated calibration tool (double arrow) and patient’s reference star (triple arrow) (with permission of Jannelli et al. atlantoaxial posterior screw fixation using intra-operative spinal navigation with three-dimensional isocentric C-arm fluoroscopy. International Orthopedics (SICOT) 46, 321–329 (2022))

In their recent surgical series, Jannelli et al. report a 100% rate of acceptable screw positioning (Gertzbein-Robbins grade A and B) using 3D fluoroscopy-based navigation (Fig. 4) [35]. Their results were consistent with a previous series from Czabanka et al. describing an accuracy of 97.9% without peri and post-operative complications [9]. Meyer et al. describe a minimally invasive, navigation-assisted percutaneous C1–C2 fixation technique for odontoid fractures and report 100% screw accuracy without intraoperative complications [47].

Similarly, navigation has favorably impacted on anterior approaches to the craniovertebral junction and odontoid fixation [4, 54, 76].

Pavlov explored the possibility of injecting contrast dye during intraoperative CT acquisition for a real-time assessment of the anatomical course—and possible variations—of the vertebral arteries and their relationship with screw trajectories [52].

Robotized navigation systems represent the new generation of computer-assisted systems with the aim of further enhancing surgical precision, which is of particular significance in the tight bony anatomy of the cervical spine. Recently, use of robotic systems has been expanded to CVJ surgery, both for the posterior [1, 16, 67] and anterior approaches [3, 48]. In their review, Lebl et al. describe the various applications of robot-assisted surgery in the cervical spine and, in particular, at the CVJ, C1 lateral mass instrumentation, C2 odontoid fracture fixation, C2 pedicle screw placement, and C2 translaminar screw fixation [43].

In 2022, Sacino et al. reported on the successful use of ExcelsiusGPS™ (Globus Medical, Inc.) for the placement of C2 pars screws to treat Hangman’s fracture and C1–C2 transarticular screws to treat degenerative atlantoaxial instability [58].

The many possibilities brought about by robotic systems, and the possibility to further enhance such systems with artificial intelligence raises several considerations: (I) the automatization of the surgical procedure allows surgeons to focus on planning, (II) procedural standardization reduces technical error, (III) such systems reduces the surgeon’s intraoperative fatigue, (IV) there is however a lack of tactile feedback, and (V) there is a possibility for image-drift. Regular verification of in vivo anatomical landmarks is therefore also required in robotic surgery.

Although free hand fluoroscopy guided screw insertion at CVJ level is considered safe in experienced hands, authors recommend the use of navigation for every spinal instrumentation at the CVJ level [35, 40], since conventional C-arm fluoroscopy comes with several shortcomings, namely, high radiation exposure, overlapping images hindering an accurate evaluation of screw trajectory, and 2D images. Computer-assisted systems, such as navigation using intraoperative 3D fluoroscopy or CT scan, address these issues and are seen as means to improve screw accuracy, reduce radiation exposure, for both patients and surgeons, and reduce implant-related neural and vascular complications. Their routine use allows for shortening of the learning curve and increasing of the surgical team performance and improving accuracy and safety in less experienced hands, like those of junior attendings and residents. Our literature analysis documented a decrease in the neurovascular complication rate from 9% to less than 1%, while the rate of screw placement accuracy was bolstered to 97–100% [35, 40, 49, 73].

From this perspective, robot-assisted CVJ surgery arises as a natural evolution and thrilling innovation that could provide a platform to integrate intraoperative image acquisition, neuronavigation, artificial intelligence, and machine learning. The development of robot-assisted surgery has been met with interest in most surgical fields and in the last years also in cranial and spinal surgery. Nevertheless, currently, there are only few but interesting reports of robot-assisted CVJ surgery but the authors believe in the continued development of this technology [35, 40, 49, 73].

Implementation of navigation and robotic techniques is particularly helpful in performing challenging CVJ surgical procedures, when it comes to the accuracy of hardware insertion in proximity of vital neural and vascular structures. Nevertheless, it must be underlined that clinically acceptable accuracy of screw insertion, which means without clinical consequences to the patient, can be achieved in experienced hands with freehand fluoroscopy guidance [2, 5, 66].

### Video-assisted surgical approaches to the CVJ

Nowadays, video-assisted procedures to the ventral skull base represent a commonly employed neurosurgical technique that has expanded the indications of endoscopic surgery. Moreover, the recent development of 3D endoscopes has allowed to overcome the 2-dimensional perception of the surgical field.

Endoscopic endonasal or transoral approaches make use of skull base anatomy to provide a direct corridor to the anterior clivus, C1 and C2, and have come to be accepted as alternatives to the more invasive microscopic transoral approaches in oncologic, traumatic, and degenerative pathologies of the CVJ, although they do carry certain disadvantages, namely, the technique’s learning curve and a restricted working channel. Moreover, transnasal approaches allow for reduction of local morbidity, as rhinolalia, dysphonia, dysphagia, and wound healing when compared to transoral approaches, and do not require a period of fasting in the postoperative period [13, 61].

In the last few years, this paradigm shift has also been clearly demonstrated in the management of CVJ pathologies [17, 24, 57, 74]. In 2022, Perrer et al. reported on their surgical series of 21 patients who underwent endoscopic endonasal odontoidectomy with favorable clinical courses at long-term follow-up, even in those cases with C1 arch preservation without posterior fixation [53], in line with the preliminary findings by Mazzatenta et al. [46].

The application of the video-assisted technologies to the anterior CVJ has given rise to multiple variations in the surgical technique: endoscopic endonasal approach, endoscopic transoral approach, robot-assisted endoscopic transoral approach, combined endoscopic transnasal and transoral approach, and endoscopic transcervical approach [69].

**Fig. 5:**
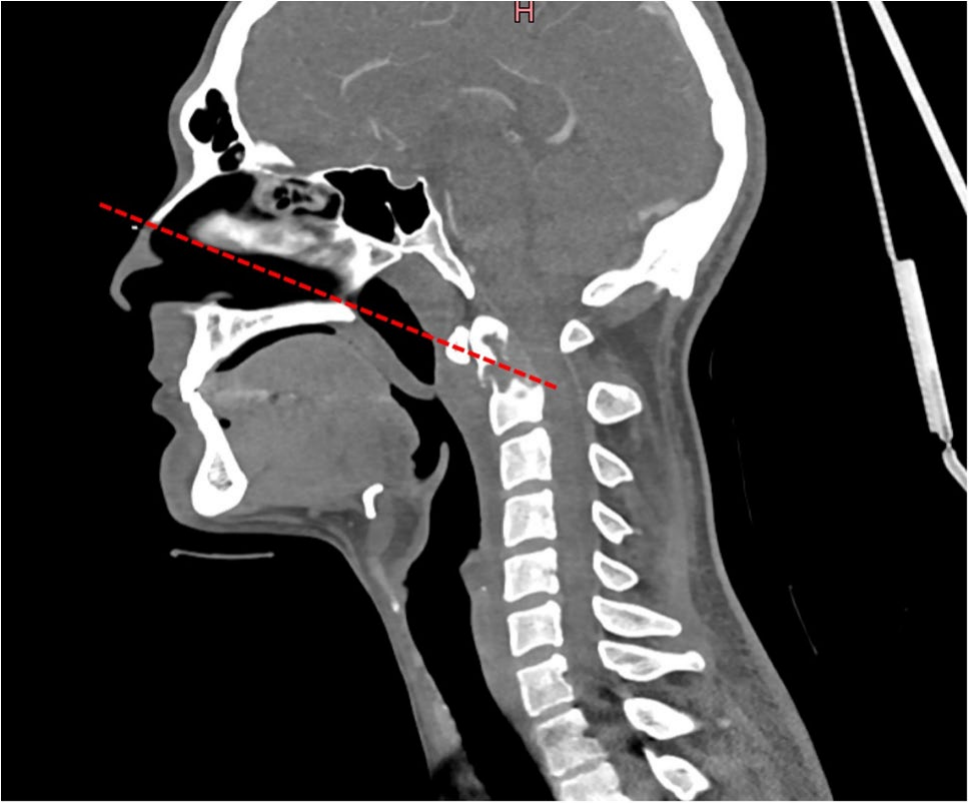
The nasopalatine line, also known as Kassam line (K line), is the line that passes between the most inferior point of the nasal bone and the posterior edge of the hard palate extending posteriorly in the midsagittal plane. The purely endoscopic endonasal procedure for craniovertebral pathologies is suitable when the junction between the dens and the body of C2 is above this line; otherwise, a combined endoscopic endonasal and transoral procedure should be preferred

When assessing pathology in the CVJ in view of an endoscopic approach, the nasopalatine line—or Kassam line (also called K line)—can be drawn connecting the most inferior point of the nasal bone to the posterior edge of the hard palate and extending posteriorly in the midsagittal plane [10, 42] (Fig. 5). When the junction of the dens and the body of C2 is above this line, the endoscopic procedure can entirely be performed through the nose; otherwise, a combined endoscopic transnasal and transoral procedure should be preferred with visualization through the nose and the handling of instruments through the mouth [11].

**Fig. 6:**
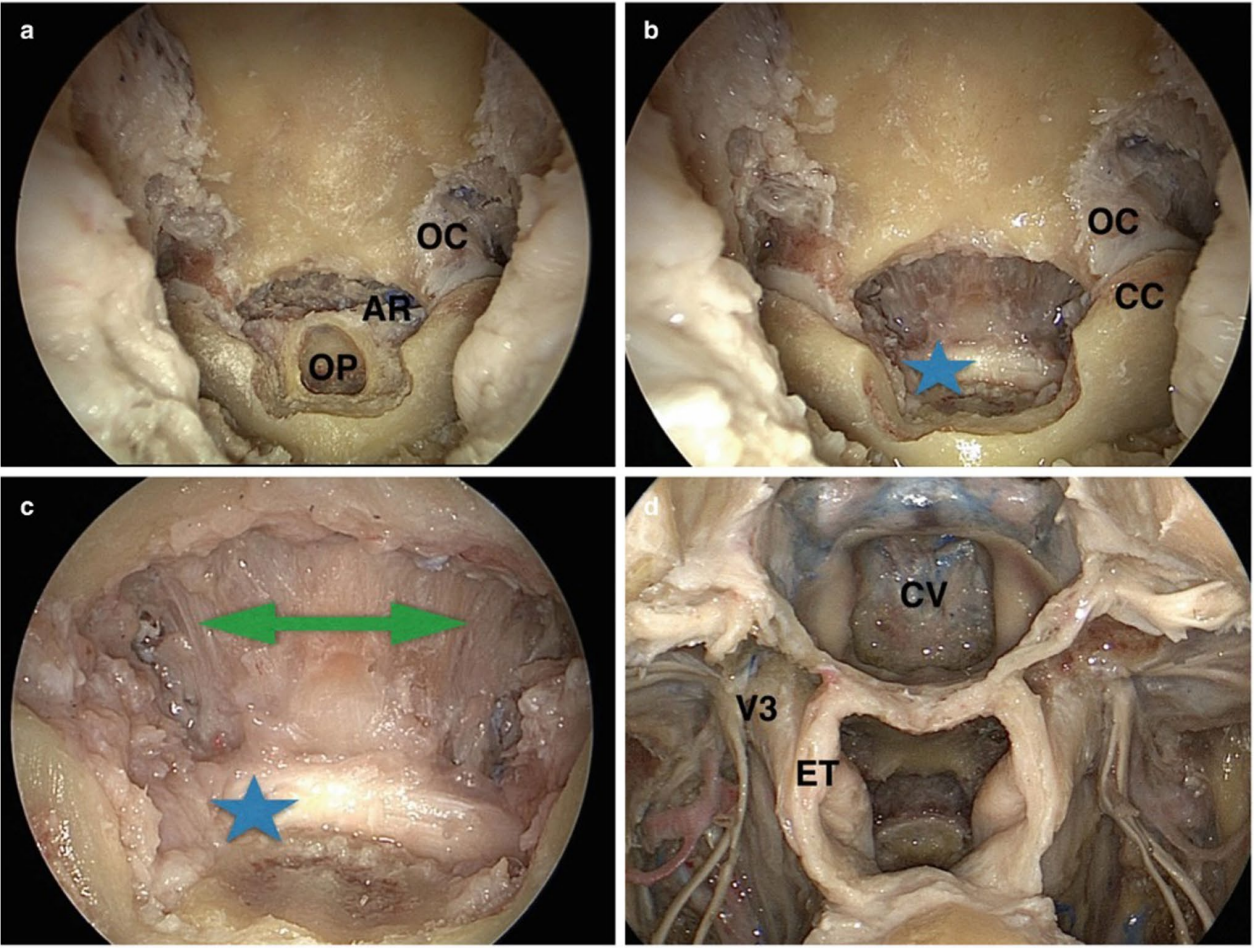
Stepwise endoscopic approach to the foramen magnum and craniovertebral junction. **a** The alar ligaments (AR) are identified, as fibrous bands attacking to the posterolateral surface of the odontoid process and ascend obliquely and laterally to dock on the alar tubercles located on the medial side of the occipital condyles (OC). Odontoidectomy starts with drilling of the central core, of the odontoid process (OP). **b, c** After dens removal, the transverse ligament (blue star) can be seen extending between the transverse tubercles on the medial side of the C1 lateral masses (CC). The tectorial membrane (green arrow) spans from the transverse ligament (blue star) to the foramen magnum. **d** Final image of the extended endonasal approach to the clivus (CV). The right and left infratemporal fossae have been exposed, showing the path of the Eustachian tube (ET) and the branches of the mandibular nerve in depth (V3) (with permission of Pacca, P., Tardivo, V., Pecorari, G., Garbossa, D., Ducati, A., Zenga, F. (2019). The Endoscopic Endonasal Approach to Craniovertebral Junction Pathologies: Surgical Skills and Anatomical Study. In: Visocchi, M. (eds) New Trends in Craniovertebral Junction Surgery. Acta Neurochirurgica Supplement, vol 125. Springer, Cham. https://doi.org/10.1007/978-3-319-62515-7_5)

The intraoperative position of the patient’s head is in slight flexion to allow a lower trajectory to the clivus and the CVJ. An extended approach is performed to expose the CVJ. After a wide posterior septostomy, the sphenoid sinus is opened, and its floor is removed with the Vidian nerve as the lateral limit. A nasopharyngeal flap is created starting just under the roof of the choana, in an inverted “U.” The upper half of the incision is carried through the nose, but in its lower extent, it is easier to raise this flap through the mouth. The lateral limits of the flap are medial to the Eustachian tube that represents an anatomical landmark for the parapharyngeal carotid artery. Finally, the attachments of the muscles are elevated off the clivus, anterior arch of C1, and dens (Fig. 6) [11].

The ongoing advancement in endoscopic skull base surgery has been transplanted to CVJ surgery to address anterior pathologies through a direct approach exploiting a close-up view of the relevant anatomy [16, 21, 53, 69]. In the past, microscopic transoral approaches proved to be effective treatments of oncologic, traumatic, and degenerative diseases of the CVJ. Nevertheless, this corridor was associated with complications and technical challenges. The introduction by the Pittsburgh group of the novel paradigm based on the endoscope and the endoscopic endonasal route allowed a straightforward access to the anterior CVJ for a 360° control of disease. Its use remains limited and technically demanding, mandating referral of such patients to specialized tertiary centers. We strongly believe that transnasal endoscopic approaches are nowadays a tremendous asset in CVJ surgery paraphernalia, having dramatically improved outcomes of patients requiring anterior decompression procedures at CVJ for tumoral and pseudo-tumoral diseases.

Increased safety at CVJ level has been obtained thanks to the implementation of endoscopic techniques for the abovementioned indications. Anterior open techniques to CVJ, e.g., transoral and transmaxillary, had been associated with an increased complication rate that is now extremely lowered by use of less invasive endoscopic guidance [17, 24, 53, 57].

### Intraoperative neuromonitoring

In CVJ surgery, spinal cord injury occurs either primarily, through direct surgical maneuvers on the spinal cord or secondarily through indirect manipulation performed on the spine, such as traction or realignment [59].

In current practice, intraoperative monitoring is reliable to predict post-operative neurological deficits [30, 44]. In view of CVJ’s proximity to important neural structures, intraoperative neurophysiological monitoring (IONM) has found applications in CVJ surgery, with muscle motor-evoked potentials (mMEPs), corticospinal motor-evoked potentials (D-wave monitoring), and somatosensory evoked potentials (SSEPs) being its most frequently used modalities.

IONM might be of help in tumoral and traumatic pathologies at CVJ bolstering the safety during all the peri-operative maneuvers. In intradural tumors, despite the limitation in D-wave utilization, IONM might provide helpful information about preservation of the neural structures. Whereas, in traumatic cases, IONM might be used during positioning of intubated and unconscious patients [30, 44].

Certain CVJ surgeries may also require monitoring of lower cranial nerve function. Cranial nerves IX, X, XI, and XII can be monitored using free-running electromyography (EMG) and the triggered EMG mapping technique. Recently, the mMEP technique has been used for the monitoring of the lower cranial nerves, where mMEPs are recorded by electrodes inserted into the muscles innervated by the lower motor cranial nerves. This technique has been shown to be more reliable than EMG [59].

In this context, we emphasize the ultimate role of intraoperative neuromonitoring in CVJ surgery reliability, which we use standardly use in our institution for all elective procedures, starting from the realignment maneuvers to patients repositioning into their bed. This allows for a timely monitoring of electrical conduction of neural elements during every phase of the surgery reducing the risk of unseen complications improving the safety throughout the whole procedure.

## Conclusions

The CVJ was formerly considered a surgically challenging region with poor postoperative outcomes. This perception has gradually changed over past decades owing to advances in surgical technique and anatomical knowledge, progress in the diagnosis and treatment of CVJ pathology, and the development of novel technologies—such as navigation, robotics and video-assistance, and neuromonitoring—that facilitate surgical approaches to the CVJ and its instrumentation, while at the same time enhancing procedural safety. Nowadays, CVJ surgery is safer and widespread to an extent not imagined only 10–20 years ago. Research on biologics and biomaterials and the use 3D-printed technology to create tailored implants have shown favorable results on fusion rates and, from there, on long-term patient outcomes. The combined continued development in these fields likely augur further progress in the safety and efficiency of the surgical and peri-operative care of the CVJ. We look forward to the next years for the further development of biologics, biomaterials, artificial intelligence, and augmented reality bringing novel boosts in the safety of CVJ surgery.
